# Alterations in genes associated with cytosolic RNA sensing in whole blood are associated with coronary microvascular disease in SLE

**DOI:** 10.1038/s41598-024-82190-4

**Published:** 2025-01-19

**Authors:** Lihong Huo, Arati Naveen Kumar, Gantsetseg Tumurkhuu, Moumita Bose, Daniel S. Berman, Daniel J. Wallace, Janet Wei, Mariko Ishimori, C. Noel Bairey Merz, Caroline Jefferies

**Affiliations:** 1 Division of Rheumatology, Department of Medicine, Cedars-Sinai Medical Center, Los Angeles, CA USA; 2 Cedars-Sinai Medical Center, Kao Autoimmunity Institute, 121 N San Vincente Blvd., Los Angeles, CA USA; 3 S. Mark Taper Foundation Imaging Center, Cedars-Sinai Medical Center, Los Angeles, CA 90211 USA; 4 Department of Cardiology, Cedars-Sinai Medical Center, Los Angeles, CA USA; 5 Barbra Streisand Women’s Heart Center, Cedars-Sinai Heart Institute, Cedars-Sinai Medical Center, Los Angeles, CA USA; 6 David Geffen School of Medicine, University of California Los Angeles (UCLA), Los Angeles, CA USA

**Keywords:** Interferon, Coronary microvascular disease, Gene expression, Systemic lupus erythematosus, Cardiovascular biology, Systemic lupus erythematosus

## Abstract

Systemic lupus erythematosus (SLE) patients are 90% women and over three times more likely to die of cardiovascular disease than women in the general population. Chest pain with no obstructive cardiac disease is associated with coronary microvascular disease (CMD), where narrowing of the small blood vessels can lead to ischemia, and frequently reported by SLE patients. Using whole blood RNA samples, we asked whether gene signatures discriminate SLE patients with coronary microvascular dysfunction (CMD) on cardiac MRI (n = 4) from those without (n = 7) and whether any signaling pathway is linked to the underlying pathobiology of SLE CMD. RNA-seq analysis revealed 143 differentially expressed (DE) genes between the SLE and healthy control (HC) groups, with virus defense and interferon (IFN) signaling being the key pathways identified as enriched in SLE as expected. We next conducted a comparative analysis of genes differentially expressed in SLE–CMD and SLE–non-CMD relative to HC samples. Our analysis highlighted differences in IFN signaling, RNA sensing and ADP-ribosylation pathways between SLE–CMD and SLE–non-CMD. This is the first study to investigate possible gene signatures associating with CMD in SLE, and our data strongly suggests that distinct molecular mechanisms underly vascular changes in CMD and non-CMD involvement in SLE.

## Introduction

Patients with SLE are at a significantly higher risk of developing cardiovascular issues, as chronic inflammation and immune dysregulation contribute to early onset atherosclerosis and damage to the heart’s blood vessels. Lupus-related heart disease may present as coronary artery disease (CAD), myocarditis, or pericarditis, contributing to long-term cardiac complications. Many women with SLE frequently report chest pain despite the absence of obstructive CAD, a condition often linked to coronary microvascular dysfunction (CMD), a form of ischemia affecting the heart’s small blood vessels. This increased cardiovascular burden makes SLE patients over three times more likely to succumb to heart disease compared to the general population^1^. CMD is a heart condition where blood flow response is impaired, resulting in reduced coronary flow reserve (CFR)^1^. This is associated with increased resistance in small blood vessels, spasms, limited myocardial perfusion reserve, and potential heart muscle ischemia, despite minimal blockage in the main heart arteries (less than 50% narrowing or a fractional flow reserve over 0.80)^2^. In terms of risk factors for CMD in the general population, notable associations have been found in women who have impaired coronary flow reserve with age, hypertension, smoking history, elevated heart rate, and low HDL^3^. Chronic inflammation also plays an important role in the pathophysiology of CMD^4^. For example, elevated levels of CRP, VCAM-1, PAI-1, vWF have been reported in CMD patients^5^. However, the molecular mechanisms underlying the development of CMD in SLE patients is unknown.

Cardiac magnetic resonance imaging (cMRI) is a non-invasive technology that allows for analysis of cardiac function, including CMD. cMRI measures determine the mass and volumes of the heart, as well as providing structural imaging of the myocardial tissue to detect fibrosis. Stress perfusion imaging facilitates the assessment of coronary blood flow, enabling the detection of ischemia and valve disorders. The myocardial perfusion index (MPRI) is a semi-quantitative method that demonstrates both sensitivity and specificity in diagnosing CMD, with an MPRI of less than 1.84 indicating CMD. Additionally, measures of left ventricular diastolic volumes and ejection fraction are employed to detect cardiac dysfunction. Previous studies by our group have demonstrated that approximately 40% (5 out of 13 in this study) of SLE patients have CMD on cMRI^6^. Interestingly in our most recent study we compared cardiac function by cMRI with clinical and inflammatory markers in a cohort of 13 SLE women. We found that left ventricular function and cardiac strain were impaired in patients with SLE compared to reference controls and correlated with increased SLICC damage index and CRP levels. However, when we analyzed cardiac function and markers of inflammation in patients with and without CMD, we observed that patients without CMD contributed more to the observed differences between SLE and reference control groups, perhaps driven by increased left ventricular mass in patients without CMD versus patients with CMD^7^. This prompted us to apply additional analyses to gain a better understanding of the molecular mechanisms underpinning CMD in SLE. RNA-sequencing (RNA-seq) offers valuable insights into the gene expression patterns within the transcriptome. In this study, our objective was to evaluate whether distinctive gene signatures can differentiate between SLE patients with CMD (SLE–CMD) and those without (SLE–non-CMD) by examining gene expression in healthy controls, SLE–CMD, and SLE–non-CMD whole blood. The findings presented in this article establish connections between peripheral biomarkers and the underlying pathobiology of SLE–CMD.

## Results

Clinical information of the study subjects is described in our previous analysis^7^. All study subjects are female, with no significant differences in age or subject characteristics (such as BMI, fasting glucose or insulin levels, and inflammatory markers such as C3, C4, CRP and ESR) between groups. Whole blood transcriptomes from the cohort of 11 SLE patients (4 with CMD and 7 without CMD) and 10 age matched healthy controls (HC) were analyzed by RNA-sequencing (RNA-seq). An overview of patient details and medications is given in Table 1.

**Table 1:** SLE patient details including age, sex (F = female), age, Disease activity (SLEDAI) and summary of medication when patients were enrolled in this study.

Record ID	Age	CMD	SLEDAI	ACE Inhibitor	Nitro-glycerin	Plaq uenil	Azathi oprine	Cell cept	Ben lysta	NSAID	Aspirin	Statin	Prednis-one (mg)
DP004	32	N	0	No	No	No	No	Yes	Yes	Yes	No	No	4
DP005	42	N	3	Yes	No	Yes	No	No	No	Yes	Yes	No	–
DP006	31	N	0	No	No	Yes	No	No	No	No	Yes	No	–
DP009	23	N	0	No	No	Yes	No	No	No	No	No	No	–
DP010	49	N	2	No	No	No	No	No	No	Yes	No	No	–
DP012	41	N	6	No	No	Yes	No	No	No	Yes	Yes	No	–
DP015	38	N	0	No	No	No	No	No	No	Yes	No	No	–
DP001	25	Y	0	No	No	Yes	Yes	No	No	Yes	No	No	10
DP002	19	Y	6	No	No	No	No	No	No	Yes	No	No	–
DP007	20	Y	0	Yes	No	No	No	Yes	No	Yes	Yes	Yes	–
DP008	33	Y	2	No	Yes	Yes	No	No	No	No	No	N0	–

Healthy controls were age and sex matched and enrolled and had blood draw on the same day as matched SLE patients.

**Fig. 1:**
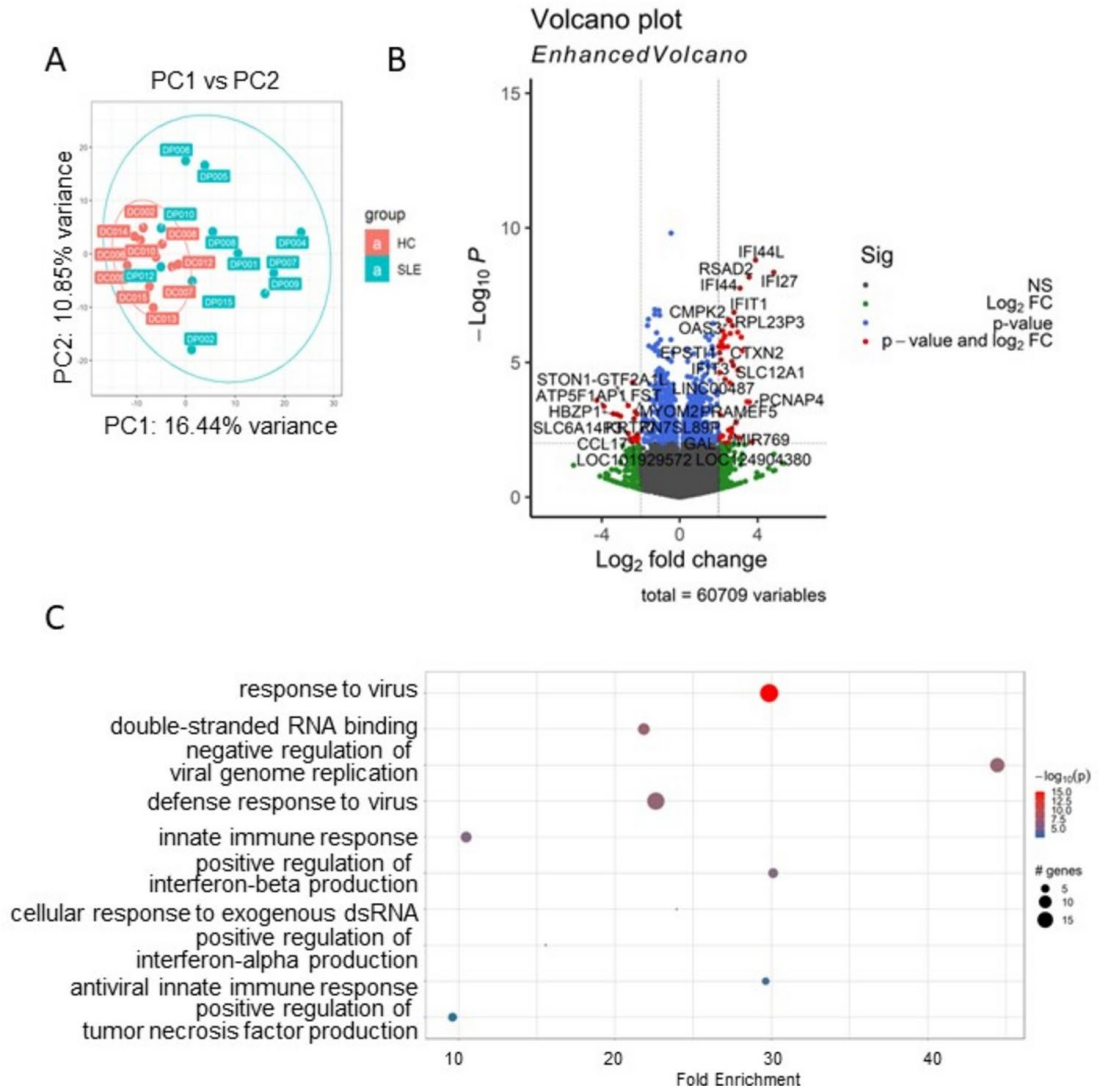
DEG and pathway analysis of SLE vs HC whole blood transcriptomes. (**A**) Principal component analysis (PCA) of whole blood transcriptomes from SLE group (n = 11) and HC group (n = 10). (**B**) Volcano plots of the gene expression comparison between SLE and HC. The horizontal axis represents the log2 (fold change) and the vertical axis represents the − log10 (*P*-value). The red plots represent the selected DEGs with fold change ≥ 2 and *p* < 0.01. (**C**) GO terms enriched in SLE patients’ samples. Genes with *P*-values < 0.05 were selected as input and enriched terms with *p*_adj_ < 0.05 were selected.

Principal Component Analysis (PCA) unveiled distinctive expression profiles between HC and SLE (Fig. 1A). After data normalization using DEseq2, preprocessing and filtering with the criteria of *p*_adj_ < 0.05, We identified 143 differentially expressed (DE) genes when comparing the SLE group and HC group. The DE gene analysis delineated a discernible molecular signature between SLE and HC, as displayed in Fig. 1B. Upon further filtering with a |log2FC|> 0.5 threshold, we identified 52 upregulated genes and 50 downregulated genes. Among the top 10 upregulated genes were IFI27, IFI44L, RSAD2, IFI44, SIGLEC1, IFIT1, SLC12A1, RPL23P3, CTXN2, ISG15, while the top 10 downregulated genes were RN7SKP227, RN7SL1, NTN4, RN7SL653P, SLC1A7, SGCD, S1PR5, KIR2DL3, LIM2, MMP23B. To gain insights into the functionality of those genes, we conducted a comprehensive Gene Ontology (GO) analysis. As expected, GO analysis results revealed SLE is significantly associated with gene enrichment of functions related to defense response to virus, type I interferon signaling pathway, and response to virus in upregulated DE genes (Fig. 1C)^8–11^.

**Fig. 2:**
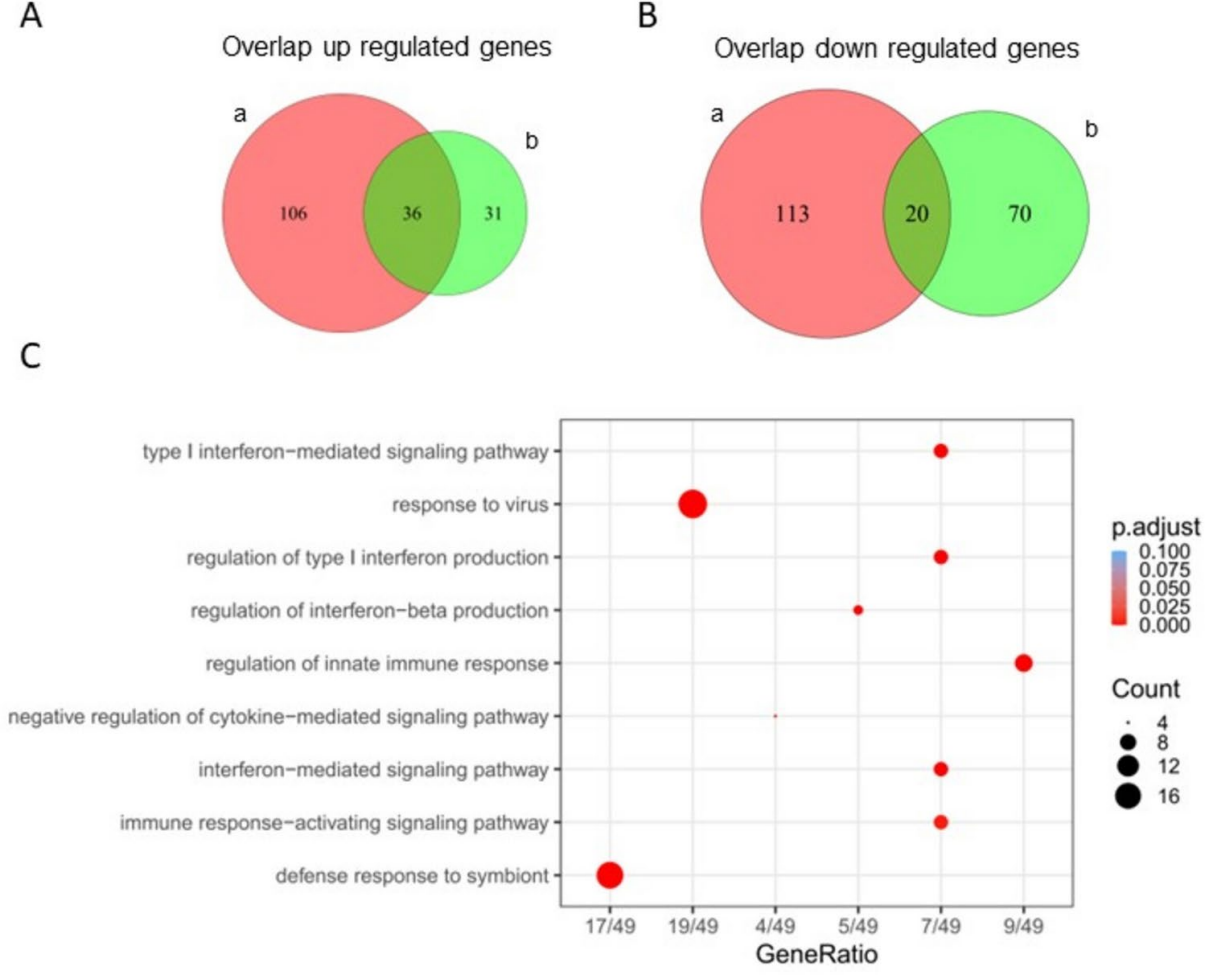
Unique gene differentially expressed in SLE–CMD and SLE–non-CMD whole blood samples using HC as reference group. (**A**, **B**) Venn diagram representing the unique or overlapping (**A**) upregulated or (**B**) downregulated genes in SLE–CMD and SLE–non-CMD when using HC as a reference group. SLE–CMD versus HC (a); SLE–non-CMD versus HC (b); DE genes were identified using DEseq2 v1.42.0 with *p*_adj_ < 0.1, log2FoldChange > 0.5 as up regulated gene cutoff, and log2FoldChang < 0.5 as down regulated gene cutoff. SLE–CMD (n = 4), SLE–non-CMD (n = 7), and HC (n = 10); (**C**) Go pathway analysis for SLE–CMD and SLE–non-CMD common genes. 60 common genes were used for this test with default filters of pvalueCutoff = 0.05, qvalueCutoff = 0.2, minGSSize = 10, maxGSSize = 500.

Next, to understand if there were differences in gene signatures between patients with or without CMD, we conducted a DE analysis comparing SLE–CMD to SLE–non-CMD. Due to the considerable variability among the samples and coupled with the fact that the dataset size is relatively small, a direct comparison between SLE groups revealed only 14 DE genes at *p*_adj_ < 0.1 (Table 2). To comprehensively understand if there were differences within whole blood transcriptomes between SLE–CMD, SLE–non-CMD, and HC groups, we employed the HC as reference and conducted a comparative analysis of commonly up regulated and down regulated genes between SLE–CMD and SLE–non-CMD. This generated datasets that comprised genes commonly upregulated or downregulated between SLE–CMD and SLE–non-CMD versus healthy control and genes uniquely up or down regulated in both patient subgroups (as shown by the Venn diagrams in Fig. 2A,B). Our investigation unveiled 36 genes that were consistently upregulated and 20 genes that were downregulated across both SLE–CMD and SLE–non-CMD groups when compared to the HC group (overlapping area of Fig. 2A,B and Table 3). Furthermore, we identified 219 genes displaying unique expression patterns between SLE–CMD and HC (left hand area of Venn diagrams in Fig. 2A,B), along with 101 unique DE genes between SLE–non-CMD and HC (right hand area of Venn diagrams in Fig. 2A,B). Analyzing the DE genes in common between SLE–CMD and SLE–non-CMD, Gene Ontology (GO) and pathway enrichment analyses indicated that these genes are clearly associated with antiviral immune responses and were primarily IFN stimulated genes (Fig. 2C).

**Table 2:** DEG between SLE–CMD and SLE–non-CMD.

Symbol	ENTREZID	log2FoldChange	*p*-value	*P*_adj_
DUS4L-BCAP29	115,253,422	7.183865	2.33E−08	0.000334
ZNF727	442,319	1.353635	3.65E−05	0.07473
PDPR	55,066	0.924705	5.01E−07	0.003591
RGPD6	729,540	0.86413	2.46E−05	0.058768
DZIP3	9666	0.671509	4.47E−05	0.080102
PYHIN1	149,628	0.637926	2.08E−05	0.05418
GPR174	84,636	0.557565	6.77E−05	0.097572
TIGIT	201,633	0.533694	6.81E−05	0.097572
C1QTNF7	114,905	− 0.94902	6.53E−05	0.097572
PTCRA	171,558	− 0.98256	4.37E−05	0.080102
JUP	3728	− 1.28328	7.27E−05	0.099248
PDE3A	5139	− 1.4114	1.74E−05	0.04974
SEPTIN7P3	646,913	− 5.27252	1.39E−05	0.044204
TBC1D3	729,873	− 9.06604	1.03E−06	0.005896

DE genes were identified using DEseq2 v1.42.0 with *p*_adj_ < 0.1. SLE–CMD (n = 4) and SLE–non-CMD (n = 7).

**Table 3:** Common genes between SLE–CMD versus HC and SLE–non-CMD versus HC.

Upregulated genes		Downregulated genes
SLC12A1	ZCCHC2	TBX21
SIGLEC1	MX1	C1orf21
OAS1	LY6E	RAB33A
OAS3	IFI27	COL6A2
OAS2	ANO5	CEP78
PRLR	FBXO39	GLB1L2
IFIH1	USP18	DLG5
IFIT3	IFIT1	PCDH1
IFI6	ISG15	CLDND2
XAF1	PLSCR1	SGCD
EPSTI1	SPATS2L	S1PR5
RSAD2	PGAP1	FCRL6
CMPK2	GRASLND	KLRC4
RTP4	CTXN2	TOGARAM2
DDX60	ZDHHC4P1	RN7SL653P
IFI44L	CCDC194	C19orf84
IFI44	HERC5	H4C12
HERC6	NKD1	

DE genes were identified using DEseq2 v1.42.0 with *p*_adj_ < 0.1, log2FoldChange > 0.5 as up regulated gene cutoff, and log2FoldChang < 0.5 as down regulated gene cutoff. SLE–CMD (n = 4), SLE–non-CMD (n = 7), and HC (n = 10).

**Fig. 3:**
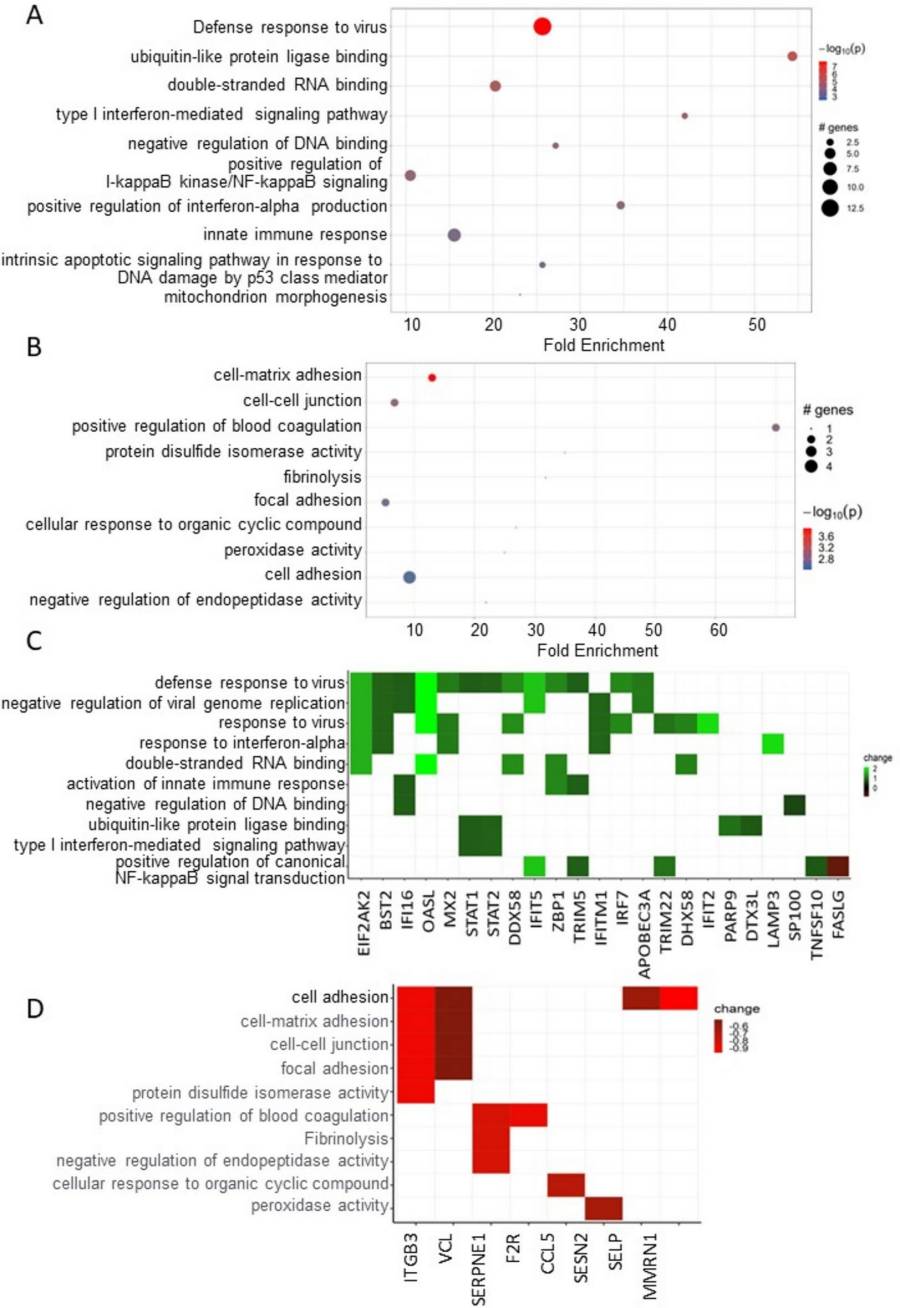
Representative DE genes in pathway analysis. (**A**, **B**) Go pathway analysis for unique genes in (**A**) SLE–CMD and (**B**) SLE-non-CMD. Genes with *p*-values < 0.05 were selected as input and enriched terms with *p*_adj_ < 0.1 were selected. SLE–CMD (n = 4), SLE-non-CMD (n = 7), and HC (n = 10); (**C**) Heatmap of SLE–CMD unique genes in the enriched GO terms. Green: up regulated; Red: down regulated; (**D**) Heatmap of unique genes in SLE-non-CMD in the enriched GO terms. Green: up regulated; Red: down regulated.

We next conducted Ontology (GO) and pathway enrichment analyses for genes unique to SLE–CMD and SLE-non-CMD patients. Sorting by *P*-value, the top GO terms of the biological process (BP), cellular component (CC) and molecular function (MF) categories are shown in Fig. 3A (SLE–CMD) and Fig. 3B (SLE-non-CMD). As GO terms for RNA sensing, double stranded (ds) RNA and single stranded (ss) RNA binding, were enriched in SLE–CMD patient samples, we further analyzed the expression of the leading-edge genes in the top GO terms (Fig. 3C). In contrast only downregulated genes in SLE-non-CMD patients were associated with any GO categories. For example, genes associated with blood coagulation, cell–cell junction, and cellular defense response were decreased in SLE-non-CMD blood samples (Fig. 3D).

**Fig. 4:**
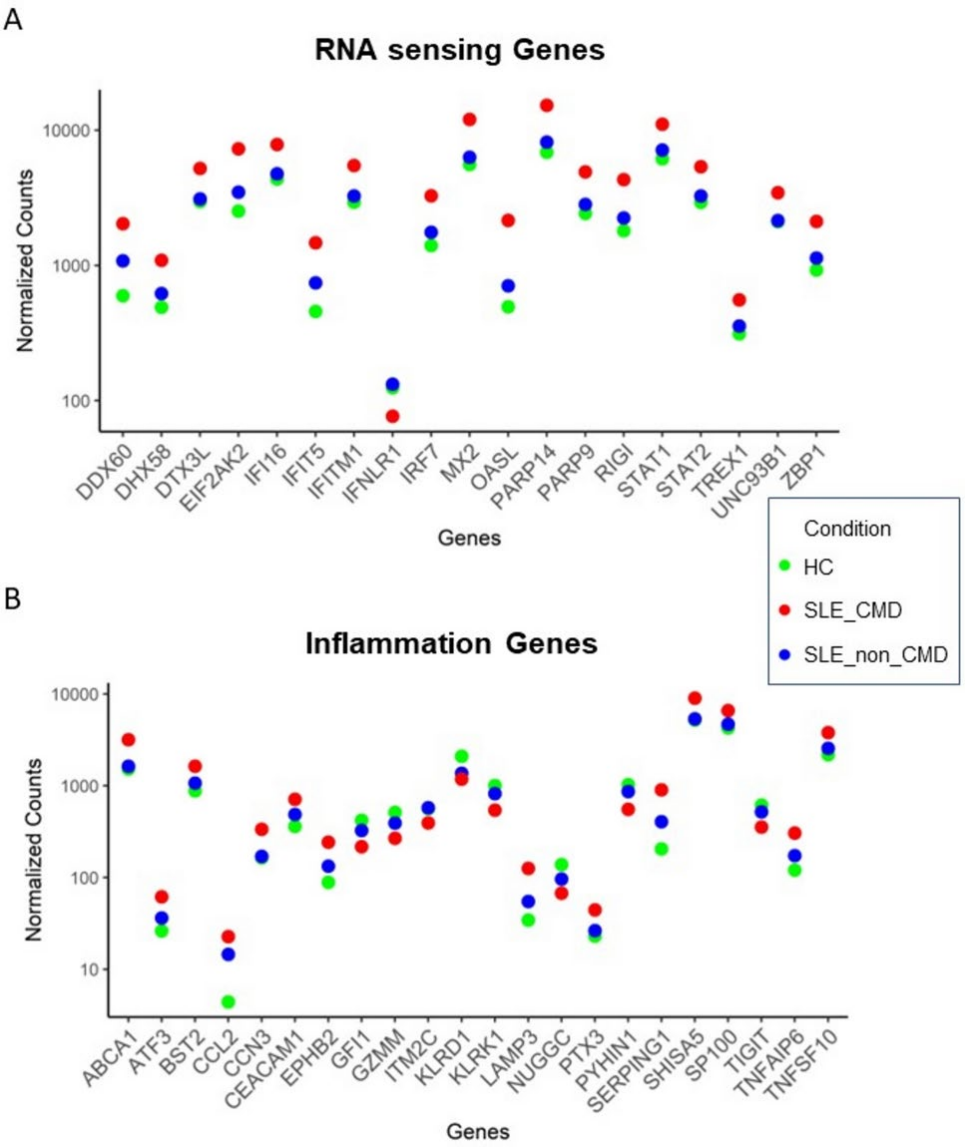
SLE–CMD unique gene signature related to RNA sensing and inflammation. (**A**, **B**) Dot plot of enrichment of RNA sensing related genes in SLE–CMD (**A**) and Dot plot of enrichment of inflammation related genes in SLE–CMD (**B**) (*P*_adj_ < 0.1, |log2FoldChange|> 0.5). SLE–CMD (n = 4), SLE-non-CMD (n = 7), and HC (n = 10).

**Fig. 5:**
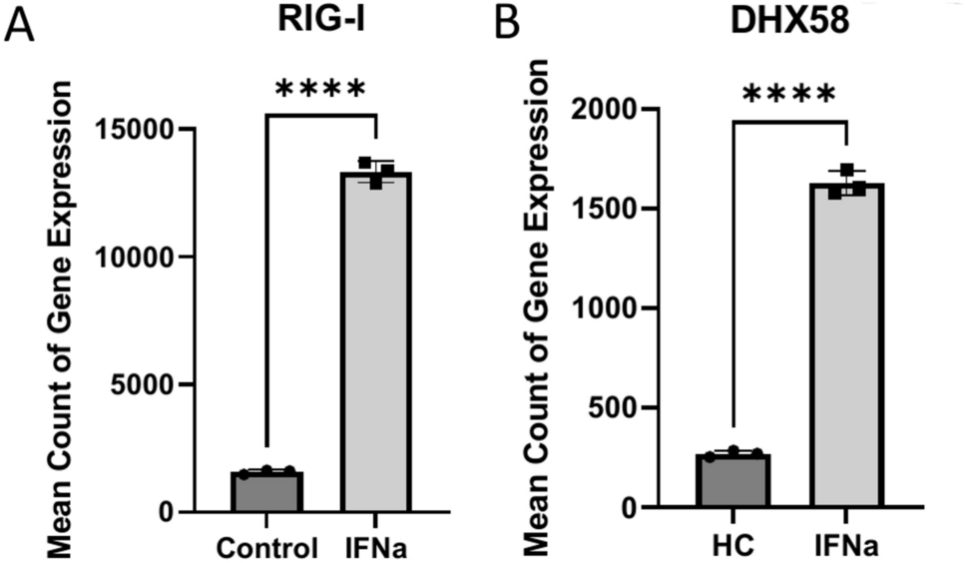
RIG-I and DHX58 expression were significantly increased in IFNα treated AC16 cells. (**A**, **B**) Bar plot of expression of RIG-I (**A**) and DHX58 (**B**) in IFNa treated Human Cardiomyocyte Cell line cells (n = 3). Statistical significance was determined by unpaired Student’s *t*-test, ****p < 0.0001.

Analyzing a subset of genes relevant to RNA sensing, we observed that a panel of genes associated with IFN signaling and response to RNA and DNA sensing such as RIGI, DDX60, DHX58 and ZBP1 were significantly increased in SLE–CMD compared to HC and SLE-non-CMD (Fig. 4A,B). In contrast, down regulated genes included the inhibitory receptor TIGIT, and markers of natural killer cells (NK) or invariant NK T cells (iNKT) KLRG1 and KLRC, suggesting that cells and pathways that might restrain cardiotoxic responses are decreased in CMD patient blood samples. Another interesting finding was the observation that IFNLR1, a component of the receptor for IFN lambda, was decreased in SLE–CMD compared to SLE-non-CMD and HC. Enzymes involved in ADP-ribosylation, a post-translational modification that regulates protein function, are also differentially upregulated in SLE–CMD patients compared to non-CMD. PARP9 and PARP14 were both increased and are part of a sub family of ADP-ribosylation enzymes that recognize mono-ADP-ribosylation (MAR) on proteins as opposed to poly-ADP-ribosylation (PAR). However, in patients with non-CMD, genes involved in coagulation, platelet activity, and cell adhesion—such as GP9, SELP (P-selectin), and ITGB3 (integrin beta 3, CD61)—were decreased specifically relative to SLE–CMD patients (Table 4). These findings suggest that RNA-sensing pathways are associated with the development of vascular changes in CMD in SLE, while reduced expression of platelet activity and coagulation-related genes is linked to the non-CMD cohort observed in our previous cardiac MRI study^13^. Interestingly we observed increased expression of RIG-I and DHX58 in human cardiomyocyte AC16 cells treated with IFNa suggesting IFN effects on cardiomyocytes may also be playing a role (Fig. 5A,B). Indeed, closer examination of the ISG between CMD and non-CMD showed higher levels of select ISGs in patients with CMD, suggesting IFN response in these patents was stronger or perhaps more prolonged (Fig. 4A). This is the first study to identify gene signatures that may associate with CMD involvement in SLE and may reveal an underlying mechanism that drives microvascular remodeling in response to cytosolic RNA/DNA sensing pathways.

**Table 4:** Gene expression changes in SLE–CMD versus non-CMD patients.

Gene	Biological pathways	Expression in SLE–CMD	Expression in non-CMD
PARP9	ADP-ribosylation (MAR)	Increased	Decreased
PARP14	ADP-ribosylation (MAR)	Increased	Decreased
GP9	Coagulation, Platelet activity	Decreased	Increased
SELP (P-selectin)	Platelet adhesion, cell adhesion	Decreased	Increased
ITGB3 (CD61)	Platelet adhesion, cell adhesion	Decreased	Increased
IFN signaling and RNA sensing genes (e.g., RIG-I, DHX58, DDX60)	RNA sensing and IFN signaling	Increased	Decreased

## Discussion

Chest pain is a common complaint among individuals with SLE even when there is no evidence of obstructive coronary artery disease (CAD)^6^. It is crucial to consider ischemic mechanisms like Coronary Microvascular Dysfunction (CMD) and coronary vasospasm during the diagnostic assessment. Given the heightened risk of cardiovascular disease (CVD) mortality and morbidity associated with SLE, stemming from inflammatory and metabolic pathophysiological processes, there is a pressing need for early identification and prevention of CVD risk factors. Hence, comprehending the molecular patterns within SLE–CMD may offer valuable insights into diagnostic methods and potential treatment approaches for clinical applications.

Endothelial dysfunction is a key driver of microvascular dysfunction and CMD. Endothelial cells (ECs) serve as a barrier between circulating blood components and the vascular wall and as such, play an important role in regulating immune responses to danger associated (such as oxLDL, heatshock proteins) and pathogen-associated molecular patterns (bacterial and viral proteins or nucleic acids) (DAMPs and PAMPs, respectively). In doing so they secrete cytokines and chemokines that regulate inflammation and immune responses, in addition to proteins that regulate vascular tone and function. Viral infection of ECs results in the activation of anti-viral signaling pathways in order to contain and eliminate the infection. One of the key ways viruses are detected by an infected cell is through recognition of viral RNA and DNA in the cytosol by RNA and DNA sensing proteins. RIG-I, MDA-5 and DHX58 (or LGP2) are three members of the RIG-I like receptors (RLRs) that are RNA helicases that recognize viral-derived dsRNA, become activated and drive the production of type I IFNs. A number of cardiotoxic agents such as 25-hydroxylcholesterol, angiotensin II and type I IFNs themselves have been shown to upregulate RIG-I expression. Together the increased expression of IFNs and IFN stimulated genes such as ISG15, CXCL10 and OAS2, are known to trigger EC dysfunction, specifically nitric oxide (NO) decreases leading to decreased vasodilation, enhanced leukocyte adhesion, inflammation, coagulation, and endothelial cell injury. However, the direct role of RIG-I-mediated IFN induction in endothelial cells is relatively unexplored. One study showed that systemic administration of a RIG-I ligand led to impaired endothelium-dependent vasodilation in mouse aorta, although whether the effects of RIG-I activation were endothelial specific in this case was not clear^12^. Another study showed that similar to the effects of another RNA sensing receptor TLR3 on fibroblast to cardiomyocyte reprogramming through NFκB, RIG-I is also involved in reprogramming of fibroblasts into cardiomyocytes through the Rig1:YY1 pathway^24^. Attenuation of DNA damage through upregulation of DNA exonuclease TREX1 in macrophages has also been demonstrated to confer cardio-protection in myocardial infarction^25^. These studies elucidate that DNA and RNA sensing pathways play important roles in response to cardiac injury across different cell types such as endothelial cells, fibroblasts and cardiomyocytes.

Our data suggest a strong association between the upregulation of RIG-I, DHX58, and other RNA/DNA sensing components such as ZBP2 in whole blood RNA samples from SLE patients with CMD, as compared to healthy controls or SLE patients with chest pain but no CMD or obstructive disease. The involvement of PARP9 and PARP14 further underscores the connection between RNA sensing pathways and CMD pathobiology. Both known regulators of RNA sensing and IFNβ induction, PARP9 amplifies type I interferon production in dendritic cells and macrophages through the PI3K/AKT3 pathway and PARP14 binds to and controls the nuclear accumulation of a subset of interferon-stimulated gene (ISG)-encoded proteins^13–15^. Our data would suggest that increased levels of genes involved in RNA sensing may contribute to enhanced expression of ISGs such as OAS2 and CXCL10 that can alter endothelial function and potentially contribute to CMD.

Additionally, our study highlights the role of adaptive immune responses in SLE–CMD. Specifically, the observations that elevated levels of NKG2D associate with SLE–CMD, suggest a role for natural killer cells in immune events contributing to CMD. Relative to this study, studies have indicated that the interaction between immune cells and cardiomyocytes via NKG2D and its ligand NKG2DL can induce cardiomyocyte death, exacerbating cardiac remodeling following myocardial infarction^16^. The reduction of the T cell specific inhibitory receptor TIGIT in SLE–CMD compared to SLE-non-CMD whole blood samples, also supports a role for dysregulated adaptive immune responses. Exhausted T cells express TIGIT and are not only a feature of tumor immunity but also of SLE and lupus nephritis in the MRL/lpr model of SLE^17^. Our finding in SLE–CMD that TIGIT levels are decreased suggests that there is a less exhausted phenotype in our cohort of SLE–CMD compared to SLE-non-CMD patients and indicates that T cell activity may contribute to microvascular dysfunction in CMD.

The potential mechanisms by which this RNA-sensing gene signature promotes CMD in SLE patients appear to involve with multiple interconnected pathways. Endothelial dysfunction, driven by interferon signaling and inflammation, appears to be one leading contributor. The upregulation of RNA sensing genes could lead to chronic IFN production, amplifying the immune response and triggering endothelial injury, NO reduction, and coagulation changes that are hallmarks of CMD. Additionally, adaptive immune dysfunction, as evidenced by altered NK and T cell receptor signaling, further complicates the endothelial landscape by promoting immune cell-mediated injury and inflammation. These processes could create a feedback loop, wherein immune activation exacerbates endothelial dysfunction, which in turn fuels further immune dysregulation, perpetuating CMD.

In conclusion this study has unveiled a compelling RNA sensing gene signature in whole blood samples of SLE patients with CMD compared to SLE patients without. Whilst our study is limited by the small samples size our signature is robust and agrees with recent literature examining the role of RIG-I and TREX1 in heart disease. While circulating microRNAs have been suggested as biomarkers for early-stage CAD, to date no biomarkers for CMD have been described. Both oxidative stress and epigenetic regulation of such pathways have previously been linked with CMD. Whether mitochondrial function and oxidative stress are contributing to mtRNA and DNA release in SLE with CMD patient immune cells is a potential mechanism driving these findings is an intriguing possibility^18,19^. Interestingly, CMD is common in patients with prior COVID infection and CMD considered a strong driver of morbidity and mortality associated with COVID-19^20,21^**.** However, whether prior viral infection drove this signature in our CMD patient cohort is not known. While our study provides valuable insights into the potential mechanisms driving CMD in SLE patients, the small sample size and the preliminary nature of this work limits the significance of the findings and highlight the need for further investigation. For example, further analysis on a larger cohort would be required to understand the relationship between prior viral infections, and the observed gene signature in SLE–CMD patients. Through larger and more comprehensive studies, we aim to validate our findings and better understand the pathophysiological processes underlying CMD and develop targeted interventions to improve cardiovascular outcomes in SLE patients.

## Materials and methods

### Patients and healthy controls

The institutional review board at Cedars-Sinai Medical Center approved this cross-sectional study, and all participants provided informed consent before joining. Participants were recruited from the lupus clinic. Inclusion criteria comprised female SLE subjects aged 37 to 57 years at baseline who experienced chest pain suspected to be angina. Exclusion criteria involved individuals with documented obstructive CAD and those with contraindications to coronary computed tomography angiography (CCTA) or cardiac magnetic resonance imaging (cMRI) as previously described^7^. SLE patients detailed information and medication are list in Table 1. Healthy controls were age and sex matched and recruited from Cedars-Sinai Medical Center. Healthy subjects are defined as no left chest pain, no autoimmune diseases nor underlying diseases. Blood samples were collected from all patients and controls in the fasting state using PAXgene blood RNA tubes.

### PAXgene RNA isolation

Blood samples were kept in PAXgene RNA tubes in -80 until ready to process. RNA was isolated using the PAXgene Blood RNA kit according to the manufacturers guidelines (PreAnalytiX). Eluted RNA was dissolved in RNase-free water. The quality and quantity of RNA were evaluated using the Agilent 2100 BioAnalyzer (Santa Clara, CA, USA).

### Cell culture and stimulation

AC16 human ventricular cardiomyocytes were cultured in complete DMEM (Life Technologies Corporation, Carlsbad, CA, USA) with 10% FBS (omega scientific, Tarzana, CA, USA) and 1% Antibiotic–Antimycotic (Gibco, Grand Island, NY, USA). At 80% confluency, cells were treated with 1000U/ml of Human recombinant IFNα (Biolegend, San Diego, CA, USA) for 24 h. Cells were then washed and lysed for RNA extraction. The quality and quantity of RNA were evaluated using the Agilent 2100 BioAnalyzer (Santa Clara, CA, USA).

### RNA sequencing

Total RNA samples were analyzed for RNA integrity on the 2100 Bioanalyzer using the Agilent RNA 6000 Nano Kit (Agilent Technologies, Santa Clara, CA) and quantified using the Qubit RNA HS Assay Kit (ThermoFisher Scientific, Waltham, MA). Three hundred ng of total RNA Total was ribodepleted using the RiboCop Depletion Kit Human/Mouse/Rat v2 (Lexogen Inc., Greenland, NH). Stranded RNA-Seq library construction was performed using the xGen Broad-Range RNA Library Prep Kit (Integrated DNA Technologies, Coralville, IA). Library concentration was measured with a Qubit fluorometer (ThermoFisher Scientific), and library size was evaluated on a 4200 TapeStation (Agilent Technologies). Multiplexed libraries were sequenced on a NovaSeq 6000 (Illumina, San Diego, CA) using 75 bp single-end sequencing. On average, approximately 50 million reads were generated from each sample.

### Bioinformatics and data analysis

Raw reads obtained from RNA-Seq were aligned to the transcriptome using STAR (version 2.5.0) /RSEM (version 1.2.25) with default parameters, using a custom human GRCh38 transcriptome reference downloaded from https://www.gencodegenes.org, containing all protein coding and long non-coding RNA genes based on human GENCODE version 33 annotation. Expression counts for each gene in all samples were normalized by a modified trimmed mean of the M-values normalization method and the unsupervised principal component analysis (PCA) was performed with DESeq2 Bioconductor package version 1.42.0 in R version 4.3. Each gene was fitted into a negative binomial generalized linear model, and the Wald test was applied to assess the differential expressions between two sample groups by DESeq2. Benjamini and Hochberg procedure was applied to adjust for multiple hypothesis testing, and differential expression gene candidates were selected with a false discovery rate less than 0.05 (Supplementary files 1-3). For functional enrichment analysis across sample groups, we conducted genes enrichment analysis using the R package “clusterProfiler v4.10.0”^22^ and “pathfindR v2.3.1^23^. The datasets have been deposited in GEO under access number GSE264125.

## Supplementary Information

Supplementary Information 1.

Supplementary Information 2.

Supplementary Information 3.

## Data Availability

Data is provided within the manuscript or supplementary information files and is openly available in Gene Expression Omnibus (GEO)] at https://www.ncbi.nlm.nih.gov/geo, reference number GSE264125.
